# The potential of β‐cell growth promotion, continued

**DOI:** 10.1111/1753-0407.13396

**Published:** 2023-04-27

**Authors:** Zachary T. Bloomgarden

**Affiliations:** ^1^ Mount Sinai Medicine/Endocrinology New York New York USA

A recent commentary in the *Journal of Diabetes* described new approaches to type 1 diabetes (T1D), pointing out that reduction in β‐cell proliferative capacity may be an underrecognized contributor to the development of T1D.^1^ A new publication describes an approach to ascertainment of β‐cell mass in 16 persons with T1D selected either for “low glucose variability” based on glycated hemoglobin (HbA1c) ≤ 7.0% and infrequent hypoglycemia with intact awareness, or “high variability” with HbA1c ≥8.5% with ≥two severe hypoglycemic events during the past year with impaired awareness. The low versus high variability groups had mean age 40 versus 37, diabetes duration 14 versus 13 years, HbA1c 6.4 versus 9.5%, and body mass index 24 versus 26 kg/m^2^. ^68^Ga‐labeled exendin positron emission tomography scanning was used as a measure of relative β‐cell mass, showing 76% greater standardized uptake values in the group with better control and less hypoglycemia, with the authors observing that this measure appeared to be a better discriminator than C‐peptide of the high versus low glucose variability groups.^2^ β‐cell mass, then, matters during the course of T1D, and there also is evidence that greater β‐cell mass has the potential to reduce the autoimmune process underlying T1D, as suggested by experiments using a cross between the nonobese diabetic mouse, a T1D model showing pancreatic islet infiltration by autoreactive immune cells with β‐cell destruction, and a liver‐specific insulin receptor knockout causing insulin resistance with β‐cell proliferation prior to onset of the autoimmune process; leading to higher insulin and C‐peptide levels with hyperplastic islets and minimal islet inflammation, with greater numbers of Treg cells.^3^

With these considerations, it is intriguing to consider understanding of β‐cell growth stemming from the elucidation of the molecular mechanism of Multiple Endocrine Neoplasia Type 1 (MEN1). In 1997, a variety of lines of investigation led to the recognition that MEN1 is caused by inactivating mutations of a gene product, subsequently named menin. Menin is a widely expressed nuclear protein present at sites of active transcription, at promoter regions of thousands of genes along with other regulatory proteins, linking histones to gene‐specific transcription factors and involved in epigenetic histone modification. Understanding of menin's role in the β‐cell has come from studies of animals with obesity and during pregnancy, physiologic states associated with expansion in β‐cell mass, which appear to be negatively regulated by menin.^4^ Transforming growth factor β may act along with menin in repressing islet protein transcription.^5^ In the β‐cell menin binds to TEAD (Transcription Enhancing factor Domain)‐1 to repress target gene expression, suppressing β‐cell proliferation.^6^ Glucagon‐like peptide‐1 may act in part to phosphorylate menin, removing its suppressive action.^7^

An animal model of type 2 diabetes (T2D) presented last year showed that daily treatment with an oral menin inhibitor, BMF‐219, led to a greater reduction in HbA1c levels than seen with liraglutide, an effect appearing to persist weeks after discontinuation of the medication.^8^ Preliminary results of a clinical trial have now been reported; 10 patients with T2D were treated for 4 weeks with BMF‐219, with baseline HbA1c 7.8%, showing substantial reduction in A1c levels persisting at 4 weeks after the agent was discontinued.^9^ Potential issues with menin inhibition would certainly need to be addressed before this agent can be used clinically. The clinical syndrome of MEN1 is associated with both benign and malignant tumors at multiple sites, including the anterior pituitary and parathyroid, and with neuroendocrine tumors also developing at other locations.^10^ Menin is a widely distributed regulatory protein, and an orally administered small molecule inhibitor might not only increase β‐cell mass but might have untoward effects in the islets as well as at other sites, and very careful evaluation of animal and then of human subject studies will be required. The promise of amelioration of an underlying cause of diabetes is, however, exciting, and we look forward to learning more about this approach in the near future.
